# QTL Mapping for RVA Profile Characteristics in a Recombinant Inbred Line Population Derived from High-Harvest-Index Yuexiangzhan Rice

**DOI:** 10.3390/plants15060880

**Published:** 2026-03-12

**Authors:** Yunyi Zhan, Zhanhua Lu, Wei Liu, Shiguang Wang, Tengkui Chen, Yongchun He, Weifeng Yang, Liting Zhang, Xiuying He

**Affiliations:** 1 Guangdong Provincial Key Laboratory of Plant Molecular Breeding, College of Agriculture, South China Agricultural University, Guangzhou 510642, China; zhanyy2001@163.com (Y.Z.);; 2 Guangdong Key Laboratory of Rice Science and Technology, Key Laboratory of Genetics and Breeding of High Quality Rice in Southern China (Co-Construction by Ministry and Province), Ministry of Agriculture and Rural Affairs, Rice Research Institute, Guangdong Academy of Agricultural Science, Guangzhou 510640, China

**Keywords:** rice, recombinant inbred line, RVA profile, QTL

## Abstract

Rapid Visco Analyzer (RVA) profile characteristics are important indicators of rice (*Oryza sativa* L.) eating quality. In this study, based on the high-density genetic linkage map constructed under the genetic background of Yuexianghzan (YXZ) and Shengbasimiao (SBSM), combined with the RVA profile characteristic data of recombinant inbred lines (RILs) grown in two environments, QTL scanning was performed using the ridge regression analysis method. A total of 59 QTLs associated with RVA profile characteristics were detected across 11 chromosomes in the two environments, with individual QTLs explaining 0.12% to 85.16% of the phenotypic variation. Moreover, 11 QTLs were repeatedly detected in two environments with large effects. The QTL located in the 1.44–1.85 Mb interval on chromosome 6 simultaneously controlled eight RVA profile characteristics and contained the cloned *waxy* (*Wx*) gene. Additionally, the intervals 20.58–20.70 Mb on chromosome 5 and 24.96–25.42 Mb on chromosome 8 were repeatedly mapped and influenced multiple RVA characteristics. Based on gene annotation information, a total of nine candidate genes (*LOC_Os05g34730*, *LOC_Os05g34830*, *LOC_Os05g34854*, *LOC_Os06g03910*, *LOC_Os06g04200*, *LOC_Os06g42720*, *LOC_Os08g39830*, *LOC_Os08g39850*, and *LOC_Os08g39860*) that directly or indirectly influence the starch synthesis pathway were identified. The results of this study lay a foundation for further map-based cloning of genes related to rice RVA profile characteristics and molecular design breeding.

## 1. Introduction

Rice (*Oryza sativa* L.) is one of the most important food crops in the world, playing a crucial role in global food security. With the breakthroughs in high-yield and stable-yield cultivation technologies of rice and the improvement of people’s living standards, rice quality has gradually become the primary factor determining market price and consumer acceptance. Rice quality includes appearance quality, milling quality, nutritional quality, and eating and cooking quality. Eating and cooking quality is not only one of the most important traits affecting rice price [1], but also a key target trait for the breeding of high-quality rice varieties. It refers to the energy required for rice cooking, as well as the characteristics of cooked rice, such as elasticity, stickiness, hardness, aroma, and taste.

The content of amylose is the primary factor responsible for significant variations in eating and cooking quality. Previous studies have typically evaluated the eating quality of rice using amylose content (AC), gelatinization consistency (GC), gelatinization temperature (GT), and taste score (TS) [2,3,4,5]. However, in practical operations, detecting these indicators is complex, time-consuming, and labor-intensive, and can only reflect quality properties from a single perspective. The Rapid Visco Analyzer (RVA) offers the advantages of short analysis time, simplicity, rapidity, and comprehensive reflection of eating and cooking quality. Consequently, it has been increasingly adopted by researchers and widely applied in rice breeding practices in China [6]. The RVA profile reflects starch pasting properties, representing changes in viscosity of starch paste during continuous heating, constant high temperature, and cooling processes. The characteristic values derived from the RVA profile include peak viscosity (PKV), hot paste viscosity (HPV), cool paste viscosity (CPV), breakdown viscosity (BDV), setback viscosity (SBV), consistency viscosity (CSV), peak time (PeT), and pasting temperature (PaT). Each RVA characteristic value shows a significant correlation with cooked rice texture, and can effectively evaluate rice eating and cooking quality [7,8]. Chen et al. found that indica rice varieties with superior eating and cooking quality exhibited higher PKV and BDV, but lower HPV, CPV and CSV [9]. Therefore, investigating the genetic mechanism of RVA profile characteristics is of great significance for the improvement of rice eating and cooking quality.

The RVA profile characteristics of rice are quantitative traits controlled by both major and minor genes, and are also the result of the combined effects of environment and genotype [10,11]. Zhang et al. [12] conducted genetic analysis of RVA profile characteristics using an F_2_ population and found that PKV, with a unimodal frequency distribution, is controlled by minor genes; HPV, CPV, SBV, and CSV, with bimodal frequency distributions, are governed by a pair of major genes and simultaneously influenced by multiple minor genes. PeT is jointly affected by two pairs of major genes and multiple minor genes. In addition, through QTL mapping of RVA characteristics using a recombinant inbred line (RIL) population, a total of 34 related QTLs were identified on chromosomes 1, 2, 3, 4, 6, 7, and 8 [12]. Bao et al. [13] utilized a doubled haploid (DH) population and discovered that, except for PKV, the other characteristics are subject to the major gene effect of *Wx* on chromosome 6. Zhang et al. [14] detected QTLs for quality traits in two environments using a backcross recombinant inbred line population, among which the stable QTLs involve *qPKV2* located in the interval R1843–S2068, *qPKV7* in R2829–R2401, *qCPV1* in C470–R1944, *qBDV4* in G264–G177, *qBDV7* in R2829–R2401, and *qSBV7* in R2829–R2401. Zhang et al. [15] constructed a chromosome segment substitution line (CSSL) population and repeatedly detected 10 stable QTLs over two years in two environments, with multiple QTLs exerting pleiotropic regulation on peak viscosity and setback viscosity. Cai et al. [16] identified a significant locus co-localized with *RAmy1A* (the gene encoding α-amylase) via a genome-wide association study in a japonica rice population with the *Wx^b^* genetic background. *RAmy1A* can affect rice eating and cooking quality by regulating starch viscosity properties. In addition, Zhao et al. [17] used varieties carrying the same *Wx^b^* allele as parents to construct a chromosome segment substitution line population for QTL mapping, eliminating the major effect of the *Wx* gene. A total of 26 repeated QTLs associated with starch synthesis-related genes (SSRGs) were detected, and multiple minor QTLs controlling starch viscosity traits near the *Wx* gene were accurately localized. To date, numerous QTLs associated with RVA profile characteristics have been mapped [12,13,14,15,16,17,18], but relatively few related genes have been cloned and reported. For example, known genes regulating starch metabolism and structure comprise *GWD1* [19], *FLO6* [20], *FLO2* [21], *du-1* [22], *GIF1* [23], and *Wx* [24]. Notably, the *Wx* gene regulates AC through its allelic variations and modulates RVA profiles via polymorphic sites including Wx-Int1 (G/T) and Wx-EX10 (C/T) [25,26]. *Wx*-Int1 (G/T) plays a dominant role in regulating PKV, BDV, and SBV, contributing 30.6%, 35.1%, and 49.7% of the phenotypic variation, respectively. While *Wx*-EX10 (C/T) mainly controls HPV, CPV, and CSV, with contribution rates of 45.8%, 58.7%, and 38.0% [25]. The *Wx^lv^* allele increases PKV, HPV, CPV and SBV and decreases BDV, whereas the *Wx^b^* allele exerts opposite effects [27]. The core of these regulatory differences lies in the alterations to starch physicochemical properties induced by *Wx* alleles. Furthermore, CRISPR/Cas9 editing of the *Wx* gene has been shown to decrease AC and modify RVA profiles, leading to improved eating quality [28], indicating that *Wx* expression level also influences RVA characteristics.

RVA profile characteristics, which are closely associated with the eating and cooking quality of rice, serve as one of the core indicators for quality evaluation [29]. Although considerable progress has been achieved in QTL mapping related to eating and cooking quality, relatively few studies have specifically focused on dissecting the QTLs underlying RVA profile characteristics to date. In this study, a RIL population (F_10_-F_11_) was used as the experimental material. This population was derived from a cross between Yuexiangzhan (YXZ) and Shengbasimiao (SBSM), two high-quality indica rice varieties from Guangdong. Then, the RVA profile characteristics of the population were measured across two seasons in 2023. Combined with a high-density bin map, QTLs controlling RVA characteristics were identified. The present work aims to explore major and stable QTLs applicable for the improvement of rice eating and cooking quality, and to provide valuable genetic information and candidate loci for further gene cloning and molecular breeding in rice. These findings offer promising genetic resources for dissecting genes related to rice eating and cooking quality, and promote marker-assisted breeding of high-quality rice.

## 2. Results

### 2.1. RVA Profile Characteristics and Wx Genotypes of Parents

**Figure 1:**
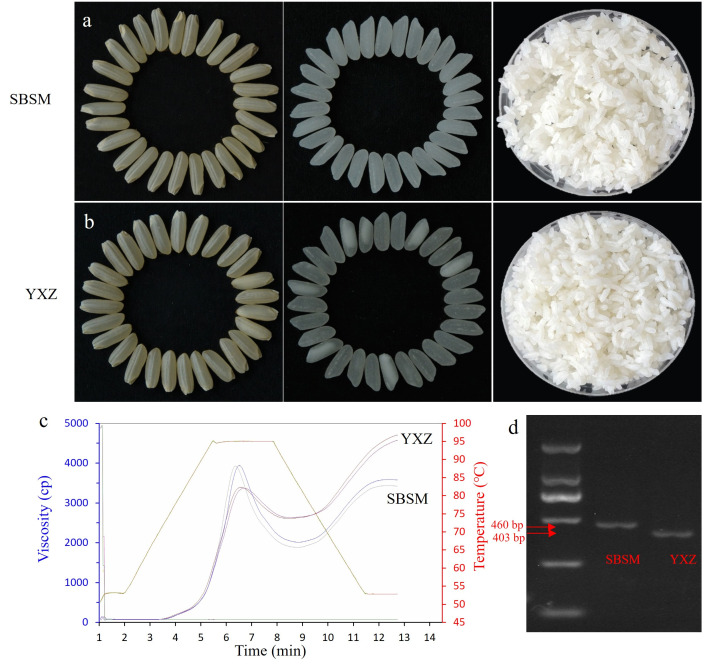
Eating and cooking quality and *Wx* genotypes of parental lines YXZ and SBSM. (**a**,**b**) Brown rice, milled rice, and cooked rice of SBSM and YXZ. (**c**) RVA profiles. (**d**) Detection of *Wx* genotypes. YXZ, Yuexiangzhan; SBSM, Shengbasimiao.

The grain of SBSM exhibited qualities such as dense, milky white and without chalkiness; the cooked rice was intact, moist, glossy, and sticky. For YXZ, it featured good transparency and a few chalky grains, and the cooked rice was intact, loose, dry, and fluffy (Figure 1). Comparison of RVA profiles between YXZ and SBSM revealed substantial differences in their pasting curves, particularly in key characteristic values including PKV, HPV, CPV, SBV, and BDV, which showed significant variations (Figure 1d). As shown in Table 1, across two environments (early and late seasons in 2023), YXZ exhibited significantly lower PKV, BDV, and PaT compared with SBSM, while the HPV, CPV, SBV, CSV, and PeT levels were significantly higher than those of SBSM. Phenotypes of all RVA traits were relatively stable across the two environments. To determine the *Wx* genotypes of the two parents, genotyping was performed using molecular markers specific to the *Wx* gene. The results showed that SBSM carries the *Wx^b^* allele, whereas YXZ harbors the *Wx^a^* allele (Figure 1d).

### 2.2. Phenotypic Analysis of RVA Profile Characteristics in the RIL Population

**Figure 2:**
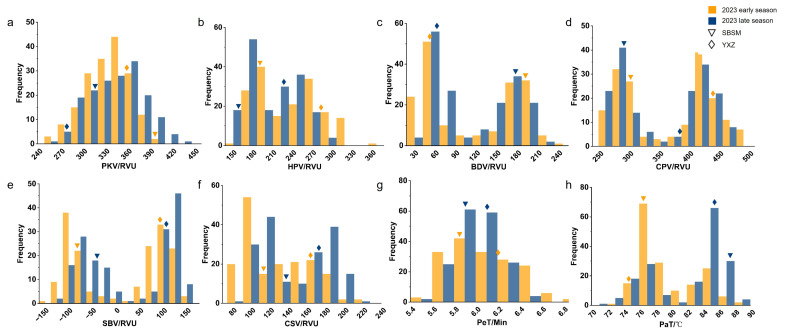
Frequency distribution of RVA profile characteristics in the RIL population. (**a**) Peak viscosity (PKV). (**b**) Hot paste viscosity (HPV). (**c**) Breakdown viscosity (BDV). (**d**) Cool paste viscosity (CPV). (**e**) Setback viscosity (SBV). (**f**) Consistence viscosity (CSV). (**g**) Pasting time (PeT). (**h**) Paste temperature (PaT). RVU, Rapid Visco Unit; Orange indicates the early season in 2023; Blue indicates the late season in 2023; YXZ, Yuexiangzhan; SBSM, Shengbasimiao; ◇ represents YXZ; ∇ represents SBSM.

To characterize the phenotypic variation in RVA profile characteristics in the RIL population, analysis was performed on eight traits. The results revealed that the mean values of all traits except PaT were intermediate between the two parents and all exhibited bidirectional transgressive segregation (Table 1). The RIL population displayed a wide range of phenotypic variations. PeT and PaT were relatively stable, while SBV, BDV, and CPV showed larger variation ranges. In addition, SBV had the highest phenotypic coefficient of variation, indicating that the genes controlling these traits are complex in number and effect. Specifically, the extremely high coefficients of variation in SBV (756.85% and 282.82%) could be explained by the nature of this derived trait. SBV values in our population ranged from −156.75 to 155.33 in the early season and −115.67 to 152.00 in the late season, with a small mean value. As the coefficient of variation is computed as the ratio of the standard deviation to the mean, expressed as a percentage, a small mean value resulted in an extremely high coefficient of variation (CV) value, even when the absolute phenotypic variation was moderate. The kurtosis values of all RVA profile traits were negative, indicating platykurtic and relatively flat distributions. Most traits were approximately symmetric across the early and late seasons; only CSV and PeT showed slight positive skewness in the early season, whereas PaT exhibited mild skewness in opposite directions between the two seasons, implying a strong environmental effect. All RVA indices exhibited a bimodal distribution except for PKV, and PeT exhibited a clear bimodal pattern (Figure 2), suggesting the presence of a major gene segregating in the population. This was confirmed by the Q-Q plot (Figures S1 and S2), which showed a characteristic *S* shape deviating from normality, indicating that the data are likely drawn from a mixture of two distributions. These results suggest that the genetic architecture of this trait may involve a major locus with a large effect, along with additional minor-effect modifiers contributing to the within-group variation.

### 2.3. Correlation Analysis of RVA Profile Characteristics

**Figure 3:**
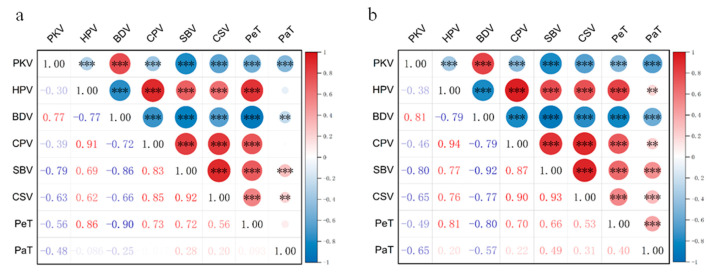
Correlations among RVA profile characteristics. (**a**) 2023 early season. (**b**) 2023 late season. Red circles indicate positive correlations, while blue circles indicate negative correlations. PKV, peak viscosity; HPV, hot paste viscosity; BDV, breakdown viscosity; CPV, cool paste viscosity; SBV, setback viscosity; CSV, consistency viscosity; PeT, peak time; PaT, pasting temperature. Values are correlation coefficients. Red/blue colors denote positive/negative correlations. Circle size indicates the correlation coefficient, the larger the circle, the higher the correlation coefficient. ** Significant at *p* < 0.01. *** Significant at *p* < 0.001.

Correlation analysis of RVA profile characteristics in the early season of 2023 showed that all indices were extremely significantly or significantly correlated with each other, except for PaT, which had no significant correlation with HPV, CPV, and PeT (Figure 3). Specifically, PKV showed an extremely significant positive correlation with BDV; the correlation coefficient reached 0.77 in the early season and 0.81 in the late season. However, PKV exhibitedextremely significant negative correlation with HPV, CPV, SBV, CSV, PeT, and PaT. HPV exhibited an extremely significant positive correlation with CPV, SBV, CSV, and PeT, and an extremely significant negative correlation with BDV. BDV showed an extremely significant negative correlation with CPV, SBV, CSV, and PeT, and a significant negative correlation with PaT. CPV showed an extremely significant positive correlation with SBV, CSV, and PeT. SBV demonstrated an extremely significant positive correlation with CSV, PaT, and PeT. CSV showed an extremely significant positive correlation with PeT and a significant positive correlation with PaT. Most correlations among the various characteristics in the late season of 2023 were consistent with those in the early season, which further confirms that RVA profiles are regulated by major-effect genes. In addition, partial correlations of RVA profile characteristics showed environmental variation. For example, the correlations between PaT and HPV, BDV, CPV, CSV, and PeT varied across seasons. These results indicate that distinct synergistic or antagonistic relationships exist among the RVA profiles’ characteristic indices, jointly regulated by both genotype and environmental factors.

### 2.4. QTL Mapping of RVA Profile Characteristics

Combined with a high-density bin map, which was used for QTL mapping of milling rice shape [30], QTL analysis was performed on the RVA profile characteristics of materials from both early and late seasons in 2023. Using a significance threshold of LOD = 3, a total of 59 QTLs were identified (Table 2), with 3 to 12 QTLs identified for each trait. The phenotypic variation explained (PVE) by a single QTL ranged from 0.12% to 85.16%.

**Figure 4:**
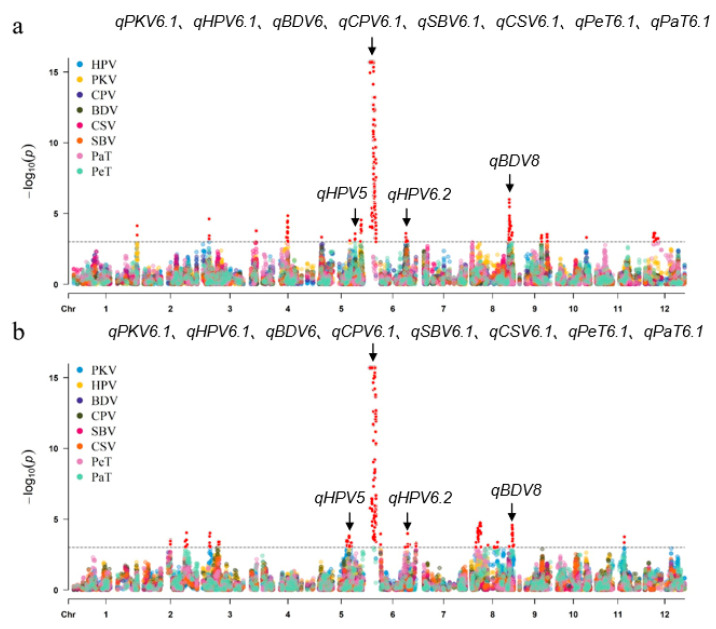
Manhattan plot of QTLs associated with RVA profile characteristics. (**a**) 2023 early season. (**b**) 2023 late season. PKV, peak viscosity; HPV, hot paste viscosity; BDV, breakdown viscosity; CPV, cool paste viscosity; SBV, setback viscosity; CSV, consistence viscosity; PeT, pasting time; PaT, paste temperature. The gray dashed line represents the threshold line of 3.

Most markers were distributed below the threshold line, with only a few significant peaks observed (Figure 4), indicating that RVA profile characteristics are coordinately regulated by major-effect QTLs and minor-effect QTLs. An extremely significant association peak was detected on chromosome 6, with its LOD value close to 15. This suggests the presence of a major-effect QTL regulating the RVA profile characteristics in this region, which has a large effect and is a key regulatory region for RVA traits. In addition to chromosome 6, significant peaks were also detected on chromosomes 5 and 8, with LOD values ranging from 5 to 8. The regions and significant trends of these peaks were highly consistent across two growing environments, indicating that these regions harbor QTLs associated with RVA phenotypes and can be further explored as potential QTLs.

A total of 12 QTLs associated with PKV were identified, distributed across chromosomes 1, 3, 4, 5, 6, 8, 9, and 11. The PVE by these QTLs ranged from 2.78% to 35.85%, with additive effect values between −6.09 and 2.15. *qPKV6.1* was mapped to the interval of 1.44–3.38 Mb on chromosome 6, which performed prominently in both the early and late seasons, accounting for 32% and 35.85% of the phenotypic variation, respectively. The PVEs of the remaining QTLs were all less than 10%, among which *qPKV4*, *qPKV5.1*, *qPKV5.2*, *qPKV5.3*, and *qPKV9* exhibited positive additive effects on PKV.

Nine QTLs linked to HPV were detected, located on chromosomes 2, 3, 5, 6, 8, and 10. Their PVE ranged from 0.12% to 65.66%, with additive effect values ranging from −6.19 to 17.22. Among these, *qHPV5*, *qHPV6.1*, and *qHPV6.2* were repositioned across both early and late seasons, distributed between 20.58 and 20.70 Mb (chromosome 5), 1.44–3.38 Mb (chromosome 6), and 24.18–25.85 Mb (chromosome 6), respectively. These three QTLs contributed the majority of the phenotypic variation in both seasons, with combined PVEs of 61.07% (early season) and 73.83% (late season). *qHPV5* and *qHPV6.1* showed positive additive effects on HPV, with the favorable alleles derived from YXZ. *qHPV6.2* showed a negative additive effect on HPV, with the favorable allele derived from SBSM. Another QTL with a relatively large effect was *qHPV8*, mapped to the interval of 24.96–25.37 Mb on chromosome 8, which explained 7.52% of the phenotypic variation in the early season.

Three QTLs controlling BDV, namely *qBDV2*, *qBDV6*, and *qBDV8*, were mapped to chromosomes 2, 6, and 8, respectively. Their PVE ranged from 2.39% to 80.57%, with additive effect values between −11.1 and 2.86. Among these, *qBDV6* and *qBDV8* were detected in both seasons, located in the intervals of 1.44–3.38 Mb (chromosome 6) and 24.96–25.42 Mb (chromosome 8), respectively. The PVE of *qBDV6* was 69.42% and 80.57% in the early and late seasons, while that of *qBDV8* was 4.41% and 2.39% in the two seasons, respectively.

A total of eight QTLs associated with CPV were identified, distributed across chromosomes 2, 3, 5, 6, and 8. Their PVE ranged from 0.12% to 79.13%, with additive effect values ranging from −6.32 to 26.54. *qCPV6.1* was mapped to the interval of 1.44–3.38 Mb on chromosome 6, which exhibited significant effects in both the early and late seasons, accounting for 62.16% and 79.13% of the phenotypic variation, respectively. The PVEs of the remaining QTLs were all less than 10%, belonging to minor-effect genes. Among these, *qCPV2*, *qCPV3.2*, *qCPV6.2*, *qCPV6.3*, and *qCPV8* showed negative additive effects on CPV.

Three QTLs regulating SBV were identified as *qSBV2*, *qSBV6.1*, and *qSBV6.2*, located on chromosomes 2 and 6, respectively. Their PVE ranged from 3.11% to 85.16%, with additive effect values between −2.87 and 15.95. Among them, *qSBV6.1* was mapped to the interval of 1.44–3.38 Mb on chromosome 6, explaining 55.04% and 85.16% of the phenotypic variation in the early and late seasons, respectively. *qSBV6.1* exhibited a distinct positive additive effect and stable expression across environments. In addition, *qSBV6.2* was only detected in the early season, contributing 19.79% of the phenotypic variation with an additive effect of 10.34. To further investigate potential interactions among these QTLs, epistasis analysis was performed using two-way ANOVA within each environment. In the early season, the interaction between *qSBV6.1* and *qSBV6.2* was not significant (p=0.266), despite significant main effects of both QTLs (p<0.001) (Table S1). Similarly, in the late season, the interaction between *qSBV2* and *qSBV6.1* was not significant (p=0.464), while their main effects were highly significant (p<0.001) (Table S2). These findings suggest that the QTLs act additively.

Four QTLs related to CSV were detected, distributed on chromosomes 3, 6, and 9. Their PVE ranged from 4.05% to 65.01%, with additive effect values between −1.61 and 5.37. *qCSV6.1* was mapped to the interval of 1.44–2.00 Mb on chromosome 6, which exhibited significant effects in both the early and late seasons, with PVEs of 51.1% and 65.01%, respectively, and showed a positive additive effect. Another QTL with a relatively large effect was *qCSV6.2*, located in the interval of 3.32–4.40 Mb on chromosome 6, which explained 15.67% of the phenotypic variation in the late season.

Ten QTLs associated with PeT were identified, distributed across chromosomes 5, 6, 8, and 9. Their PVE ranged from 1.01% to 40.19%, with additive effect values between −0.03 and 0.07. *qPeT6.1* was mapped to the interval of 1.44–3.38 Mb on chromosome 6. It was consistently detected in both the early and late seasons and displayed a positive additive effect, with PVEs of 40.19% and 37.06%, respectively. Another QTL with a relatively large effect was *qPeT8.1*, which was located within the interval of 25.02–26.98 Mb on chromosome 8, accounting for 7.9% of the phenotypic variation in the early season.

A total of ten QTLs controlling PaT were detected, distributed on chromosomes 2, 4, 6, 8, and 12. The PVE ranged from 2.58% to 9.99%, with additive effect values ranging from −0.26 to 0.37. *qPaT6.1*, consistently detected in both the early and late seasons, was mapped to the interval of 1.44–1.85 Mb on chromosome 6, with PVEs of 9.99% and 8.44% in the two seasons, respectively. Another QTL with a relatively large effect was *qPaT4*, located in the interval of 15.90–17.24 Mb on chromosome 4, which explained 7.96% of the phenotypic variation in the early season.

### 2.5. Analysis of QTL Stability and Co-Localized for Multiple Traits

Most QTLs were detected only in a single environment, while 11 QTLs were repeatedly identified across both environments. The QTLs exhibited considerable stability with substantial effects, particularly *qPKV6.1*, *qHPV5*, *qHPV6.1*, *qHPV6.2*, *qBDV6*, *qBDV8*, *qCPV6.1*, *qSBV6.1*, *qCSV6.1*, *qPeT6.1*, and *qPaT6.1*. A comparison of the mapping results for different traits revealed 12 intervals that simultaneously control multiple traits (Table 3). Specifically, *qPKV6.1*, *qHPV6.1*, *qBDV6*, *qCPV6.1*, *qSBV6.1*, *qCSV6.1*, *qPeT6.1*, and *qPaT6.1* were co-localized in the 1.44–1.85 Mb interval on chromosome 6. The cloned *Wx*, a major gene controlling amylose content and regulating RVA profile characteristics [24], is located within this major QTL interval.

The three traits HPV, CPV, and PeT were found to be co-localized in two intervals on chromosome 6, 24.35–25.85 Mb and 30.20–31.24 Mb. The 3.99–4.20 Mb interval on chromosome 3 simultaneously controls PKV, HPV, and CPV, while the 9.88–10.94 Mb interval on the same chromosome co-regulates HPV and CPV. The 27.34–28.76 Mb interval on chromosome 5 has been demonstrated to regulate both PKV and PeT. PKV and PaT were found to be co-localized in the 16.66–18.23 Mb interval on chromosome 8, whereas the 25.02–25.36 Mb interval on chromosome 8 was found to regulate HPV, CPV, PeT, and BDV simultaneously.

### 2.6. Prediction of Candidate Genes for Major Effect QTLs

Based on the Rice Genome Annotation Project database (https://rice.uga.edu/), candidate genes associated with rice eating quality traits within the QTL interval identified through stable detection were screened, analyzed, and summarized. We screened all functional genes in 4 QTLs, *qHPV5* (Chr 5, 20.58–20.70 Mb), *qHPV6.1* (Chr 6, 1.44–1.85 Mb), *qHPV6.2* (Chr 6, 24.18–25.85 Mb), and *qBDV8* (Chr 8, 24.96–25.42 Mb), and selected eating quality candidate genes based on their function. A total of nine genes directly or indirectly involved in starch synthesis pathways were screened based on gene annotation information (Table 4). Among them, five genes were successfully cloned, including *LOC_Os05g34730*, *LOC_Os05g34830*, *LOC_Os05g34854*, *LOC_Os06g03910*, and *LOC_Os06g04200*. The remaining four uncloned genes were *LOC_Os08g39850*, *LOC_Os08g39830*, *LOC_Os08g39860*, and *LOC_Os06g42720*.

*LOC_Os05g34730* (*SERF1*), an ERF transcription factor responsive to salt stress, has been demonstrated to directly regulate the expression of RPBF and GBSSI, thereby affecting starch synthesis and grain filling [31]. Additionally, *SERF1* has been demonstrated to play a role in the processes of carbohydrate accumulation and stomatal movement, which may influence photosynthetic efficiency and subsequently impact starch synthesis [32]. *LOC_Os05g34830* encodes a NAC transcription factor that participates in ABA signal transduction [33]. *LOC_Os05g34854* (*OsGA20ox4*), a gene encoding GA20 oxidase involved in gibberellin biosynthesis. Previous studies have shown that plant hormones such as ABA, IAA, and GA affect the activity of starch synthases and regulate starch accumulation [34,35]. *LOC_Os06g03910* encodes *OsNUDX14*, a NUDIX hydrolase. *OsNUDX14* is related to rice chalkiness, as knockout of *OsNUDX14* in transgenic lines increases chalkiness rate and degree, elevates alkali spreading value, and slightly reduces viscosity [36]. *LOC_Os06g04200* is the *Wx* gene encoding granule-bound starch synthase (GBSS), a major regulatory gene for amylose synthesis [37,38].

*LOC_Os06g42720* encodes an amino acid transporter. Studies have confirmed that amino acid transporters affect grain storage protein content and amylose content [39]. *LOC_Os08g39850* has been found to be annotated as a lipoxygenase associated with chloroplast precursors, which can inhibit chloroplast photochemical activity and participate in cell membrane metabolism [40]. *LOC_Os08g39830* is an ethylene signal regulator that may indirectly affect the expression of starch synthesis-related genes or regulate relevant physiological processes through involvement in the ethylene signaling pathway, thereby influencing starch synthesis. *LOC_Os08g39860* is functionally annotated as a homolog of *Os8bglu27*-β-glucosidase, similar to the exoglucanase *Os4bglu12*. *Os4bglu12*-encoded exoglucanase is closely related to cell wall metabolism [41].

## 3. Discussion

### 3.1. The Major Regulatory Role of the Wx Gene

In this study, the locus located in the 1.44–1.85 Mb interval on chromosome 6 was repeatedly identified in both environments and simultaneously affected eight RVA characteristics, representing a major-effect QTL controlling RVA profile traits. Alignment with the Rice Genome Annotation Project database revealed that the cloned amylose gene *Wx* is located within this interval. We employed PCR amplification combined with restriction enzyme digestion to genotype the GT→TT mutation as the molecular marker. The results showed that SBSM carries the *Wx^b^* haplotype, while YXZ has the *Wx^a^* haplotype (Figure 1d). This allelic difference provides a molecular basis for the distinct RVA profiles observed between the two parents. Specifically, SBSM exhibited significantly higher PKV and BDV than YXZ, but lower HPV, CPV, SBV, CSV, and PeT. These phenotypic differences are consistent with the known relationship between RVA characteristics and eating quality [42]. The higher PKV and BDV, combined with lower HPV, CPV, SBV, CSV, and PeT observed in SBSM, suggest that this parent possesses superior eating quality, characterized by higher stickiness, softer texture, and lower retrogradation tendency. In contrast, YXZ tends to produce firmer cooked rice with moderate stickiness. In the present study, this locus showed an extremely high phenotypic variation explanation (the PVE of *qSBV6.1* was 85.16%) on RVA, suggesting that this locus acts as a major-effect QTL for the corresponding RVA trait. The mapping population was derived from a cross between materials carrying *Wx^a^* and *Wx^b^* alleles, resulting in a typical segregating population for the major *Wx* gene. Although RVA-related traits are generally considered to be polygenically inherited, major QTLs with large effects are not unexpected in such populations. Additionally, bimodal distributions were observed for HPV, BDV, CPV, SBV, CSV, and PaT in the RIL population, which is presumably attributed to the presence of the *Wx^a^* and *Wx^b^* haplotypes in the population. While the *Wx* gene is a strong candidate in the 1.44–1.85 Mb interval on chromosome 6 based on its known function, future fine-mapping, gene silencing, or overexpression studies are required to confirm whether the observed effects are due to true pleiotropy or tight linkage.

In addition to the major QTL for RVA traits located in the *Wx* region on chromosome 6, several other major and stable QTLs outside the *Wx* locus were identified in the present study. These included *qHPV5* (Chr5, 20.58–20.70 Mb) significantly associated with HPV; *qHPV6.2* (Chr6, 24.35–25.85 Mb) significantly associated with HPV, CPV and PeT; and *qBDV8* (Chr8, 25.02–25.36 Mb) significantly associated with HPV, CPV, BDV and PeT. We have carefully compared these QTLs with those reported in previous studies on rice RVA and eating quality traits. The results showed that *qHPV5* and *qHPV6.2* have not been reported in previous studies, suggesting that they are novel and stable genetic loci for regulating rice paste viscosity properties. All these loci were stably expressed across environments and independent of the *Wx* gene, providing new and important genetic resources for the genetic improvement of rice eating and cooking quality.

### 3.2. Comparison of Quality Genes Among QTLs Mapped in This Study

The QTLs on chromosome 6 (*qPKV6.1*, *qHPV6.1*, *qBDV6*, *qCPV6.1*, *qSBV6.2*, *qPeT6.1*, and *qPaT6.2*) overlap with the *OsSSI* gene, which encodes starch synthase I. *OsSSI* is involved in amylopectin synthesis; reduced expression of *OsSSI* increases amylose content, leading to poor eating quality [43]. The 30.20–31.24 Mb locus on chromosome 6, which co-regulates HPV, CPV, and PeT, harbors the cloned *Du13*/*TL1* genes. The C_2_H_2_ zinc finger protein encoded by *Du13* affects the splicing efficiency of *Wx^b^*; the loss of *Du13* drastically reduces the splicing efficiency of the first intron of *Wx^b^*, thereby impacting the accumulation and activity of OsGBSSI protein and lowering amylose content [44]. *qPKV8.1*, located in the 4.22–6.19 Mb interval on chromosome 8, overlaps with the cloned *OsSSIIIa*/*Flo5* gene, which is involved in the synthesis of long chains of amylopectin. *SSIIIa*-RNAi affects the activity and expression of other key starch synthases (e.g., GBSS, SSI, and BEIIa), resulting in increased amylose content [45]. Additionally, under high-temperature stress, loss of *SSIIIa* enhances the sensitivity of grain chalkiness to high temperatures during the filling stage [45]. The interval containing *qPeT8.1* includes the *OsISA1* gene, which exerts a significant influence on starch synthesis and endosperm development. Transgenic plants with knockout of *OsISA1* show significantly reduced amylose and amylopectin contents in the endosperm, along with significantly increased sugar content and starch gel consistency [46]. The *qPeT8.1* and *qPaT8.4* exhibit an overlap with the *OsMADS7*, which has been demonstrated to regulate *Wx* expression and its response to high-temperature environments [47]. *qPKV5.1*, in the 18.02–19.83 Mb interval on chromosome 5, contains the *OsPPDKB*/*FLO4* gene that encodes pyruvate orthophosphate dikinase (PPDK), which is involved in starch synthesis and storage processes. The *flo4* mutation leads to a significant reduction in total starch and amylose contents [48]. The interval containing *qPKV5.3* includes the *OsPDCD5* gene; the loss of *OsPDCD5* increases head rice rate and gel consistency, while decreasing amylose content [49]. *qCPV3.2*, located in the 9.88–10.94 Mb interval on chromosome 3, overlaps with the *OsFAD7* gene that regulates unsaturated fatty acid synthesis. *OsFAD7* promotes an increase in α-linolenic acid content in grains [50]. Higher unsaturated fatty acid content in milled rice is associated with better eating quality [51]. The interval harboring *qCSV3* in the 35.12–35.12 Mb region on chromosome 3 contains the cloned *GPA3* gene related to grain protein synthesis. The *gpa3* mutant exhibits reduced grain filling rate and forms floury endosperm with low amylose content and high protein and lipid contents [52]. The *qPaT2* interval on chromosome 2 includes the *OsMADS6*/*AFG1* gene; the loss of *AFG1* impairs grain filling, resulting in decreased amylose content, increased protein and soluble sugar contents, and elevated pasting temperature, thereby affecting rice eating quality [53]. However, these genes are considered preliminary candidates based on their genomic location, but their functional relevance requires further validation through haplotype analysis and expression profiling in future studies.

In summary, the RILs, constructed using YXZ and SBSM as parental lines, demonstrate significant advantages in identifying QTLs associated with rice RVA-related quantitative traits. This has been fully validated through the aforementioned analysis of quality genes within the mapped QTLs. Furthermore, the QTLs identified in this population highly overlap with a series of cloned genes closely associated with rice eating quality, covering multiple key metabolic pathways including starch synthesis, fatty acid synthesis, and grain protein synthesis. This indicates that this RIL population possesses rich genetic diversity for traits related to rice eating quality. Furthermore, the ability to identify minor-effect QTLs in the presence of *Wx^a^* and *Wx^b^* demonstrates the comprehensive detection capability of the RIL for complex QTLs that govern eating quality traits. In summary, the RILs from YXZ and SBSM are a reliable source of genetic information for investigating the molecular mechanisms that determine rice eating quality.

## 4. Materials and Methods

### 4.1. Experimental Materials

The test material was a RIL population of 189 lines, derived from YXZ (accession no.: Guangdong Rice Approval 1998001) and SBSM (accession no.: Guangdong Rice Approval 2005002) via single-seed descent [54]. Both YXZ and SBSM are high-quality indica rice varieties in South China. YXZ was once a control variety in the national regional trials, with cooked rice characterized by a tight, white, and glossy appearance; a rich fragrance; and a slightly hard texture, but relatively poor viscoelasticity [55]. SBSM has translucent grains, a strong rice aroma, and excellent eating quality [56]. The high-density genetic map of the RIL population contains 2412 bins, with lengths ranging from 30 kb to 3.0 Mb, an average interval of 0.99 cM, and a total length of 2376.46 cM [30].

### 4.2. Planting of Materials

The F_10_ and F_11_ generations of the RIL population, along with their parent plants, were planted at the Baiyun Experimental Base of Guangdong Academy of Agricultural Sciences, Guangzhou, Guangdong Province (113.44° E, 23.39° N, altitude 14.1 m) during the early and late seasons of 2023, respectively. Sandy loam was the dominant soil type in the experimental field, which contained 0.73 g·kg^−1^ total nitrogen, 0.74 g·kg^−1^ total phosphorus, and 63.6 g·kg^−1^ total potassium. For the first season, seeds were sown on 27 February 2023, 30-day-old seedlings were transplanted on 29 March, and harvesting was conducted on 23 July. For the second season, seeds were sown on 22 July 2023, 15-day-old seedlings were transplanted on August 6, and harvesting was performed on 12 November. All field management practices were consistent with local conventional field management.

### 4.3. Determination of RVA Profiles

After seed maturation, seeds were naturally air-dried, stored at 10 °C for 90 days, then processed into brown rice and milled rice. The milled rice was ground into powder, passed through an 80-mesh sieve, and dried at 45 °C for later use. Rice starch RVA profiles were determined using a Rapid Visco Analyzer (RVA 4500, Perten Instruments, Hägersten, Sweden). A 3 g sample of rice flour was weighed into an aluminum cylinder, mixed with 25 mL of ddH_2_O, and stirred with a paddle to ensure uniformity. The mixture was stirred with a paddle to ensure uniform mixing of rice flour and water. The aluminum cylinder was placed in the RVA instrument for a heating and cooling cycle, which lasted 12.5 min, including: heating to 95 °C, holding at 95 °C for 2.5 min, cooling to 50 °C, and holding at 50 °C for 1.4 min. The Thermal Cycle for Windows (TCW) software (RVA 4500 Software Package) was used for image acquisition and data export. The primary parameters obtained included peak viscosity (PKV/RVU), hot paste viscosity (HPV/RVU), cool paste viscosity (CPV/RVU), peak time (PeT/min), and pasting temperature (PaT/°C). Breakdown viscosity (BDV/RVU) was calculated as PKV-HPV, consistency viscosity (CSV/RVU) as CPV-HPV, and setback viscosity (SBV/RVU) as CPV-PKV. Each sample was tested in triplicate, and the average value was used.

### 4.4. QTL Mapping

Genotyping of the parental lines and 184 RILs was performed using genotyping-by-sequencing (GBS) on the Illumina HiSeq 2500 platform. Sequencing reads were aligned to the *Oryza sativa* Nipponbare reference genome. After SNP calling and filtering, high-quality SNPs were collected and used to define bin blocks with a sliding window method. A high-density bin-based genetic linkage map was constructed using R/qtl software (R 4.2.x) [30]. To control the false-positive rate, correction for multiple comparisons was conducted during QTL detection. Ridge regression analysis was used to reduce multicollinearity and further minimize false-positive QTLs, ensuring the reliability of the mapping results. A LOD threshold of 3 was set to identify significant QTLs, and the phenotypic variation explained by each QTL was calculated. The detected QTLs were named following the principles proposed by McCouch et al. [57]. To detect epistatic interactions between QTLs identified within the same environment, two-way analysis of variance (ANOVA) was performed. The ANOVA was conducted using the aov function in R, and significance was declared at *p* < 0.05.

### 4.5. DNA Extraction, PCR Amplification, and Restriction Enzyme Digestion of PCR Products

Fresh rice leaves were used for genomic DNA extraction using the CTAB method [58]. Primers were designed based on the reference genome sequence, and their specificity was analyzed using the NCBI website (https://www.ncbi.nlm.nih.gov/tools/primer-blast/index.cgi?LINK_LOC=BlastHome, accessed on 20 June 2025). Referring to the primers designed by Cai et al. [59], the upstream primer (5′-GCTTCACTTCTCTGCTTGTG-3′) and downstream primer (5′-ATGATTTAACGAGAGTTGAA-3′) were synthesized by Hangzhou Youkang Biotechnology Co., Ltd. The PCR reaction system was 20 μL, containing 1 μL DNA, 10 μL 2 × Phanta UniFi Master Mix (Dye Plus, Nanjing Vazyme Biotech Co., Ltd., Nanjing, China, P526-02), 0.8 μL of each forward and reverse primer (10 μM), and 7.4 μL ddH_2_O. The reaction program was as follows: pre-denaturation at 98 °C for 30 s, followed by 32 cycles of denaturation at 98 °C for 10 s, annealing at 60 °C for 10 s, and extension at 72 °C for 15 s, with a final extension at 72 °C for 5 min. The annealing temperature varied according to the base content of the primers. After amplification, the PCR products were digested with the restriction enzyme Acc I. The digestion system (20 μL total volume) contained 10 μL of PCR product, 1 μL of Acc I enzyme, 2 μL of 10 × M Buffer, and 7 μL of ddH_2_O. The mixture was vortexed, incubated at 37 °C for 1 h, and then subjected to 30 g/L agarose gel electrophoresis for the detection of digestion products. After electrophoresis, the gel was observed and photographed using a gel imaging system.

### 4.6. Data Analysis

Microsoft Excel and Origin 2025 were used for phenotypic analysis of RVA profile characteristics and frequency distribution histogram plotting for the parents and RIL population. Origin 2025 was used to analyze the correlations among RVA profile characteristics and generate correlation heatmaps. The Kolmogorov–Smirnov test was used to verify the normal distribution of population data. Excel was used to calculate the range, coefficient of variation, skewness, and kurtosis. Spearman’s method was used to calculate correlation coefficients.

## 5. Conclusions

A total of 189 lines from the F_10_ and F_11_ RIL population derived from YXZ × SBSM were used as materials for QTL analysis. Combined with a high-density bin map, 59 QTLs associated with RVA profile characteristics were detected across two environments, with individual QTLs explaining 0.12% to 85.16% of the phenotypic variation. *qHPV5*, *qHPV6.1*, *qHPV6.2*, and *qBDV8* were repeatedly detected across both environments. Among these, the major-effect locus *qHPV6.1* influences eight RVA characteristics, contains two candidate genes within its interval, and co-localizes with the cloned *Wx* gene—a key gene in the starch synthesis pathway. *qHPV5* and *qHPV6.2* affect HPV, containing three and one candidate genes, respectively. *qBDV8* influences BDV and harbors three candidate genes within its interval. Other QTLs also contain genes related to rice eating quality that have been cloned. On this basis, the functions of candidate genes can be further verified, providing new genetic resources and a scientific basis for mining genes related to rice eating quality and breeding high-quality rice varieties.

## Figures and Tables

**Table 1:** Phenotypic values of RVA profile characteristics in parents and populations.

Trait	Time	Parents		RIL Population				
		YXZ	SBSM	Mean ± SD	Range	CV (%)	Skewness	Kurtosis
PKV (RVU)	early	350.5	395.5 **	354.73 ± 35.29	271.00~446.58	9.95	−0.04	−0.57
	late	280.33	327.21 **	326.03 ± 29.88	248.92~391.75	9.17	−0.29	−0.43
HPV (RVU)	early	284.5	195.42 **	238.42 ± 47.20	161.67~361.50	19.80	0.25	−1.13
	late	216.29	159.25 **	210.38 ± 39.86	152.17~299.17	18.95	0.25	−1.29
BDV (RVU)	early	66	200.08 **	116.32 ± 67.02	27.08~236.00	57.62	0.13	−1.76
	late	64.04	167.96 **	115.66 ± 57.81	40.25~217.75	49.99	0.18	−1.65
CPV (RVU)	early	449.83	314.75 **	367.41 ± 73.89	262.00~496.25	20.11	0.00	−1.63
	late	386.46	290.25 **	358.19 ± 71.85	254.33~477.50	20.06	−0.02	−1.69
SBV (RVU)	early	99.33	−80.75 **	12.68 ± 95.95	−156.75~155.33	756.85	−0.13	−1.76
	late	106.13	−36.96 **	32.16 ± 90.95	−115.67~152.00	282.82	−0.16	−1.73
CSV (RVU)	early	165.33	119.33 **	128.99 ± 34.67	79.25~219.75	26.88	0.49	−0.97
	late	170.17	131.00 **	147.82 ± 35.63	91.83~222.83	24.10	0.07	−1.48
PeT (min)	early	6.2	5.87 **	6.05 ± 0.30	5.53~6.87	5.01	0.43	−0.70
	late	6.04	5.80 **	5.97 ± 0.19	5.47~6.47	3.26	0.16	−0.38
PaT (℃)	early	75.6	77.05 **	79.59 ± 3.59	73.15~89.00	4.51	0.73	−0.68
	late	84.70	85.90 **	82.35 ± 4.67	71.50~88.30	5.67	−0.67	−1.11

YXZ, Yuexiangzhan; SBSM, Shengbasimiao; PKV, peak viscosity; HPV, hot paste viscosity; BDV, breakdown viscosity; CPV, cool paste viscosity; SBV, setback viscosity; CSV, consistency viscosity; PeT, peak time; PaT, pasting temperature; RVU, Rapid Visco Unit; CV, Coefficient of Variation. ** Significant at *p* < 0.01.

**Table 2:** QTLs for RVA profile characteristics in early and late season.

Trait	Chr	Interval (Mb)	QTL	2023 First Season			2023 Second Season			Reported Genes
				*p* Value	Effect	PVE (%)	*p* Value	Effect	PVE (%)	
PKV	1	41.77–42.92	*qPKV1*	7.30 × 10^−5^	−2.30	3.19	-	-	-	-
	3	3.99–5.89	*qPKV3*	-	-	-	9.42 × 10^−5^	−1.12	3.90	-
	4	15.51–17.27	*qPKV4*	1.42 × 10^−5^	2.15	6.71	-	-	-	-
	5	18.02–19.83	*qPKV5.1*	-	-	-	3.16 × 10^−4^	0.92	4.53	*OsPPDKB*
	5	21.30–22.68	*qPKV5.2*	-	-	-	4.57 × 10^−4^	0.97	3.33	-
	5	27.07–28.83	*qPKV5.3*	1.05 × 10^−4^	1.89	2.78	-	-	-	*OsPDCD5*
	**6**	**1.44–3.38**	***qPKV6.1***	**2.00 × 10^−16^**	**−6.19**	**32.00**	**2.00 × 10^−16^**	**−3.64**	**35.85**	***Wx, OsSSI***
	6	26.27–27.35	*qPKV6.2*	-	-	-	6.20 × 10^−4^	−1.10	3.38	-
	8	4.22–6.19	*qPKV8.1*	-	-	-	1.74 × 10^−5^	−1.23	6.34	*OsSSIIIa*
	8	16.66–18.23	*qPKV8.2*	-	-	-	4.20 × 10^−4^	−0.93	3.43	-
	9	14.60–15.23	*qPKV9*	3.49 × 10^−4^	1.81	2.22	-	-	-	-
	11	17.74–19.20	*qPKV11*	-	-	-	1.76 × 10^−4^	−1.02	4.02	-
HPV	2	16.40–17.86	*qHPV2*	-	-	-	6.49 × 10^−4^	−1.86	2.98	-
	3	3.99–4.33	*qHPV3.1*	2.38 × 10^−5^	5.05	0.12	-	-	-	-
	3	9.31–10.95	*qHPV3.2*	-	-	-	5.70 × 10^−4^	−1.66	1.67	-
	5	**20.58–20.70**	***qHPV5***	**7.89 × 10^−4^**	**3.91**	**5.49**	**1.50 × 10^−4^**	**1.56**	**3.69**	**-**
	6	**1.44–3.38**	***qHPV6.1***	**2.00 × 10^−16^**	**17.22**	**48.51**	**2.00 × 10^−16^**	**9.66**	**65.66**	***Wx, OsSSI***
	6	**24.18–25.85**	***qHPV6.2***	**2.50 × 10^−4^**	**−4.38**	**7.07**	**1.04 × 10^−4^**	**−2.00**	**4.48**	**-**
	6	29.49–31.24	*qHPV6.3*	-	-	-	4.77 × 10^−4^	−2.17	3.23	*OsFD2*
	8	24.96–25.37	*qHPV8*	9.84 × 10^−7^	−6.09	7.52	-	-	-	-
	10	19.59–20.76	*qHPV10*	4.87 × 10^−4^	4.53	0.60	-	-	-	-
BDV	2	16.40–18.16	*qBDV2*	-	-	-	2.94 × 10^−4^	1.92	3.09	-
	**6**	**1.44–3.38**	***qBDV6***	**2.00 × 10^−16^**	**−11.10**	**69.42**	**2.00 × 10^−16^**	**−8.74**	**80.57**	***Wx, OsSSI***
	**8**	**24.96–25.42**	***qBDV8***	**3.84 × 10^−5^**	**2.86**	**4.41**	**8.41 × 10^−4^**	**1.59**	**2.39**	**-**
CPV	2	16.46–17.86	*qCPV2*	-	-	-	3.56 × 10^−4^	−4.32	3.36	-
	3	3.99–4.20	*qCPV3.1*	6.11 × 10^−4^	5.23	0.12	-	-	-	-
	3	9.88–10.94	*qCPV3.2*	-	-	-	3.99 × 10^−4^	−3.74	1.77	*OsFAD7*
	5	1.57–2.89	*qCPV5*	4.65 × 10^−4^	5.61	1.61	-	-	-	-
	**6**	**1.44–3.38**	***qCPV6.1***	**2.00 × 10^−16^**	**26.54**	**62.16**	**2.00 × 10^−16^**	**24.15**	**79.13**	***Wx, OsSSI***
	6	24.03–25.84	*qCPV6.2*	7.52 × 10^−4^	−5.55	4.35	-	-	-	-
	6	30.20–31.24	*qCPV6.3*	-	-	-	5.53 × 10^−4^	−4.87	2.94	*OsFD2*
	8	24.96–25.36	*qCPV8*	9.37 × 10^−5^	−6.32	2.54	-	-	-	-
SBV	2	16.40–18.16	*qSBV2*	-	-	-	5.87 × 10^−4^	−2.87	3.11	-
	**6**	**1.44–2.65**	***qSBV6.1***	**2.00 × 10^−16^**	**15.95**	**55.04**	**2.00 × 10^−16^**	**14.12**	**85.16**	***Wx***
	6	2.76–4.40	*qSBV6.2*	2.00 × 10^−16^	10.34	19.79	-	-	-	*OsSSI*
CSV	3	34.99–35.91	*qCSV3*	1.66 × 10^−4^	−1.61	4.05	-	-	-	*GPA3*
	**6**	**1.44–2.00**	***qCSV6.1***	**2.00 × 10^−16^**	**5.37**	**51.10**	**2.00 × 10^−16^**	**4.48**	**65.01**	***Wx***
	6	3.32–4.40	*qCSV6.2*	-	-	-	1.60 × 10^−14^	2.33	15.67	-
	9	18.44–19.49	*qCSV9*	2.91 × 10^−4^	−1.41	2.87	-	-	-	-
PeT	5	23.62–24.85	*qPeT5.1*	2.67 × 10^−4^	0.02	5.54	-	-	-	-
	5	27.34–27.85	*qPeT5.2*	9.11 × 10^−4^	−0.02	1.55	-	-	-	-
	5	27.99–28.76	*qPeT5.3*	2.78 × 10^−5^	−0.02	1.65	-	-	-	-
	**6**	**1.44–3.38**	***qPeT6.1***	**2.00 × 10^−16^**	**0.07**	**40.19**	**2.00 × 10^−16^**	**0.03**	**37.06**	***Wx, OsSSI***
	6	23.12–24.03	*qPeT6.2*	-	-	-	9.50 × 10^−4^	−0.01	2.65	-
	6	24.35–25.84	*qPeT6.3*	-	-	-	8.65 × 10^−4^	−0.01	3.43	-
	6	28.12–31.24	*qPeT6.4*	-	-	-	7.87 × 10^−4^	−0.01	2.41	*OsFD2*
	8	25.02–26.98	*qPeT8.1*	1.68 × 10^−6^	−0.03	7.90	-	-	-	*OsISA1*, *OsMADS7*
	8	2.99–3.12	*qPeT8.3*	-	-	-	4.33 × 10^−4^	−0.01	5.28	-
	9	14.60–15.38	*qPeT9*	9.35 × 10^−4^	−0.02	1.01	-	-	-	-
PaT	2	27.46–29.36	*qPaT2*	-	-	-	8.99 × 10^−5^	−0.21	6.49	*AFG1*
	4	15.90–17.24	*qPaT4*	8.16 × 10^−4^	−0.15	7.96	-	-	-	-
	**6**	**1.44–1.85**	***qPaT6.1***	**4.09 × 10^−6^**	**0.27**	**9.99**	**4.21 × 10^−7^**	**0.37**	**8.44**	***Wx***
	6	2.93–4.40	*qPaT6.2*	-	-	-	3.84 × 10^−5^	0.28	2.75	*OsSSI*
	8	10.95–12.92	*qPaT8.1*	-	-	-	6.73 × 10^−4^	0.17	2.70	-
	8	14.41–16.06	*qPaT8.2*	-	-	-	8.80 × 10^−4^	0.17	2.58	-
	8	16.66–18.48	*qPaT8.3*	-	-	-	9.05 × 10^−4^	0.17	2.90	-
	8	25.95–27.82	*qPaT8.4*	-	-	-	2.58 × 10^−5^	−0.26	4.38	*OsMADS7*
	8	27.91–28.26	*qPaT8.5*	-	-	-	6.62 × 10^−4^	−0.23	2.66	-
	12	5.66–7.46	*qPaT12*	2.41 × 10^−4^	−0.17	6.38	-	-	-	-

PKV, peak viscosity; HPV, hot paste viscosity; BDV, breakdown viscosity; CPV, cool paste viscosity; SBV, setback viscosity; CSV, consistence viscosity; PeT, pasting time; PaT, paste temperature; Chr, Chromosome; QTL, quantitative trait locus; PVE (%), phenotypic variance explained by a given QTL; Effect, additive effect (the positive value means that YXZ allele increases the trait value); - represents none; bold QTLs indicate those detected in both seasons and represent stable loci.

**Table 3:** Co-localized QTLs for multiple traits.

Chr	Interval (Mb)	Trait	QTL
2	16.46–17.86	HPV, CPV, SBV	*qHPV2*, *qCPV2*, *qSBV2*
3	3.99–4.20	PKV, HPV, CPV	*qPKV3*, *qHPV3.1*, *qCPV3.1*
	9.88–10.94	HPV, CPV	*qHPV3.2*, *qCPV3.2*
4	15.90–17.24	PKV, PaT	*qPKV4*, *qPaT4*
5	27.34–28.76	PKV, PeT	*qPKV5.3*, *qPeT5.2*, *qPeT5.3*
6	1.44–1.85	PKV, HPV, BDV, CPV, SBV, CSV, PeT, PaT	*qPKV6.1*, *qHPV6.1*, *qBDV6*, *qCPV6.1*, *qSBV6.1*, *qCSV6.1*, *qPeT6.1*, *qPaT6.1*
	3.32–4.40	SBV, CSV, PaT	*qSBV6.2*, *qCSV6.2*, *qPaT6.2*
	24.35–25.85	HPV, CPV, PeT	*qHPV6.2*, *qCPV6.2*, *qPeT6.3*
	30.20–31.24	HPV, CPV, PeT	*qHPV6.3*, *qCPV6.3*, *qPeT6.4*
8	16.66–18.23	PKV, PaT	*qPKV8.2*, *qPaT8.3*
	25.02–25.36	HPV, CPV, PeT, BDV	*qHPV8*, *qCPV8*, *qPeT8.1*, *qBDV8*
9	14.60–15.23	PKV, PeT	*qPKV9*, *qPeT9*

PKV, peak viscosity; HPV, hot paste viscosity; BDV, breakdown viscosity; CPV, cool paste viscosity; SBV, setback viscosity; CSV, consistence viscosity; PeT, pasting time; PaT, paste temperature; Chr, Chromosome; QTL, quantitative trait locus.

**Table 4:** Functional annotations of candidate genes.

Chromosome	Gene Accession Number	Gene Functional Annotation	Location (Mb)
5	*LOC_Os05g34730*	Ethylene-responsive transcription factor ERF020	20.60
5	*LOC_Os05g34830*	No apical meristem protein	20.68
5	*LOC_Os05g34854*	Gibberellin 20-oxidase	20.69
6	*LOC_Os06g03910*	Hydrolase, NUDIX family, domain-containing protein	1.57
6	*LOC_Os06g04200*	Starch synthase	1.77
6	*LOC_Os06g42720*	Amino acid transporter	25.69
8	*LOC_Os08g39830*	Ethylene-insensitive 3	25.21
8	*LOC_Os08g39850*	Lipoxygenase, chloroplast precursor	25.22
8	*LOC_Os08g39860*	Os8bglu27-β-glucosidase homolog, similar to Os4bglu12 exoglucanase	25.25

## Data Availability

The original contributions presented in this study are included in the article. Further inquiries can be directed to the corresponding authors.

## Supplementary Materials

The following supporting information can be downloaded at: https://www.mdpi.com/article/10.3390/plants15060880/s1, Figure S1. Q-Q plot of the RVA characteristic values in 2023 early season. (a) peak viscosity (PKV). (b) hot paste viscosity (HPV). (c) breakdown viscosity (BDV). (d) cool paste viscosity (CPV). (e) setback viscosity (SBV). (f) consistence viscosity (CSV). (g) pasting time (PeT). (h) paste temperature (PaT). Figure S2. Q-Q plot of the RVA characteristic values in 2023 late season. (a) peak viscosity (PKV). (b) hot paste viscosity (HPV). (c) breakdown viscosity (BDV). (d) cool paste viscosity (CPV). (e) setback viscosity (SBV). (f) consistence viscosity (CSV). (g) pasting time (PeT). (h) paste temperature (PaT). Table S1. Epistasis analysis of qSBV6.1 and qSBV6.2 in 2023 early season. Table S2. Epistasis analysis of *qSBV2* and *qSBV6.1* in 2023 late season.
