# Supplementary material for: QTL Mapping for RVA Profile Characteristics in a Recombinant Inbred Line Population Derived from High-Harvest-Index Yuexiangzhan Rice
Source: Plants (Basel). 2026 Mar 12;15(6):880. doi: 10.3390/plants15060880 (PMC13029979; doi:10.3390/plants15060880)
Supplement: Supplementary file 1 [file plants-15-00880-s001.zip › plants-4152663-supplementary.pptx]

## Slide 1
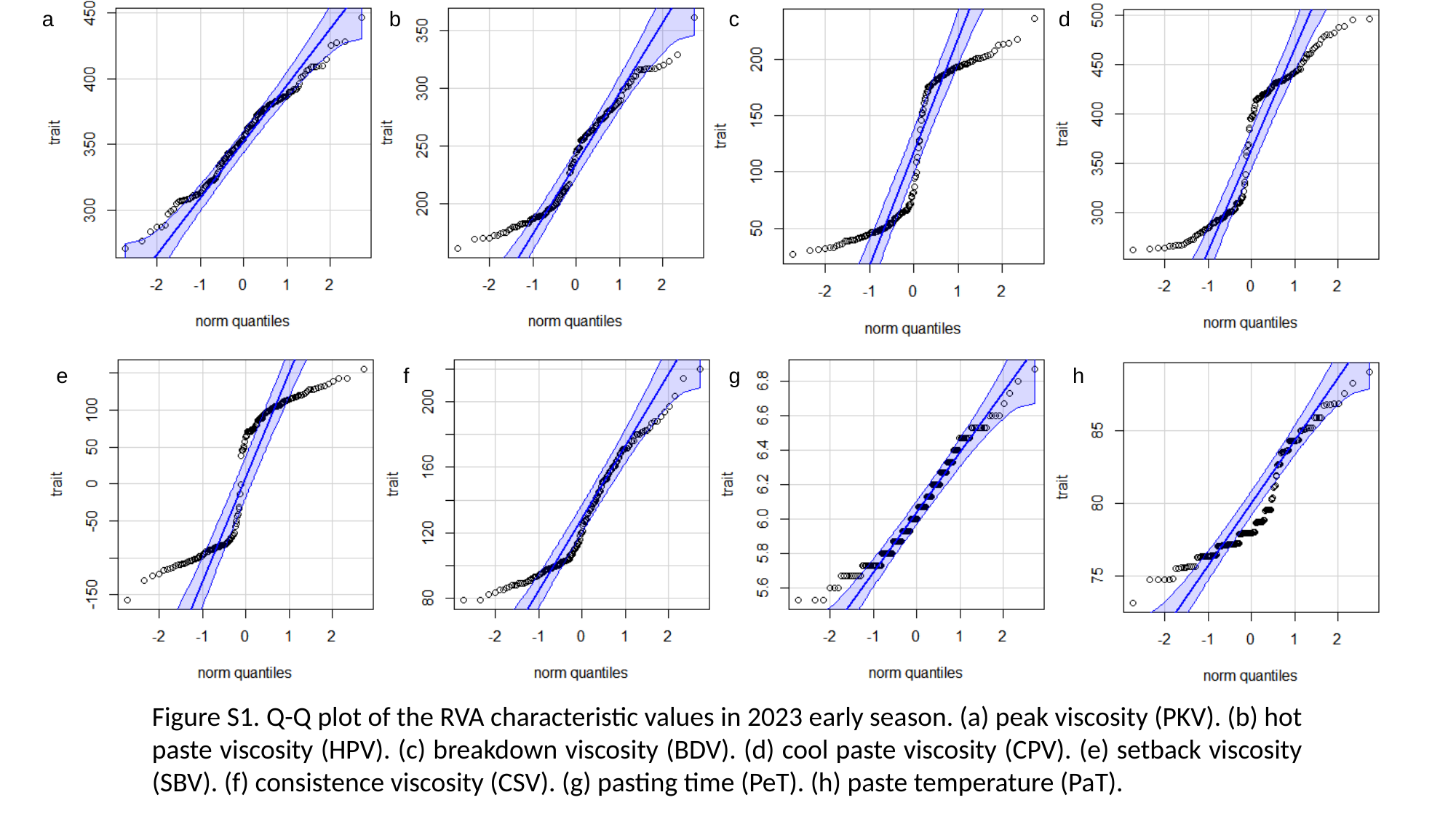

a
b
c
d
e
f
g
h
Figure S1. Q-Q plot of the RVA characteristic values in 2023 early season. (a) peak viscosity (PKV). (b) hot paste viscosity (HPV). (c) breakdown viscosity (BDV). (d) cool paste viscosity (CPV). (e) setback viscosity (SBV). (f) consistence viscosity (CSV). (g) pasting time (PeT). (h) paste temperature (PaT).

## Slide 2
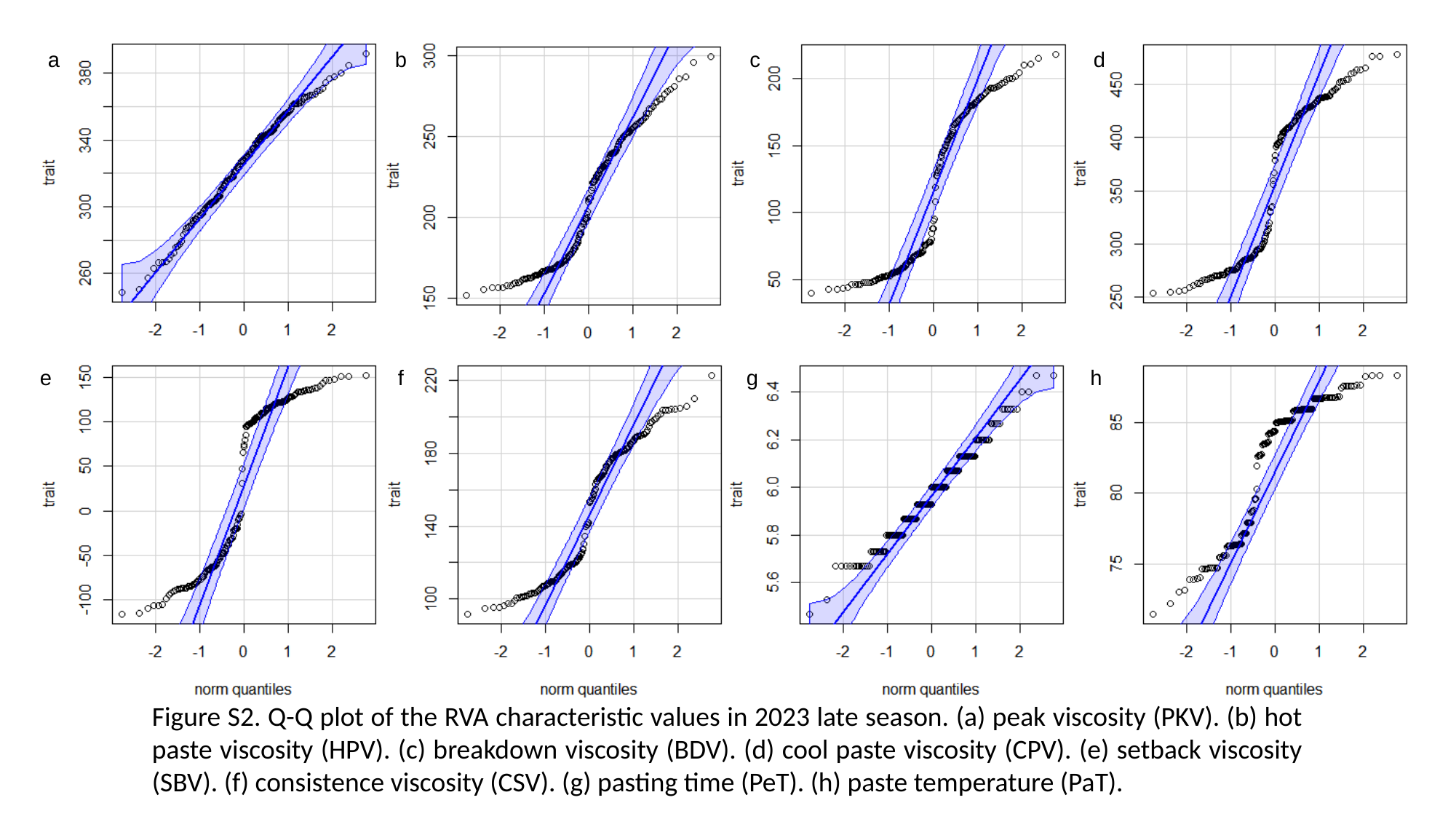

a
b
c
d
e
f
g
h
Figure S2. Q-Q plot of the RVA characteristic values in 2023 late season. (a) peak viscosity (PKV). (b) hot paste viscosity (HPV). (c) breakdown viscosity (BDV). (d) cool paste viscosity (CPV). (e) setback viscosity (SBV). (f) consistence viscosity (CSV). (g) pasting time (PeT). (h) paste temperature (PaT).

## Slide 3
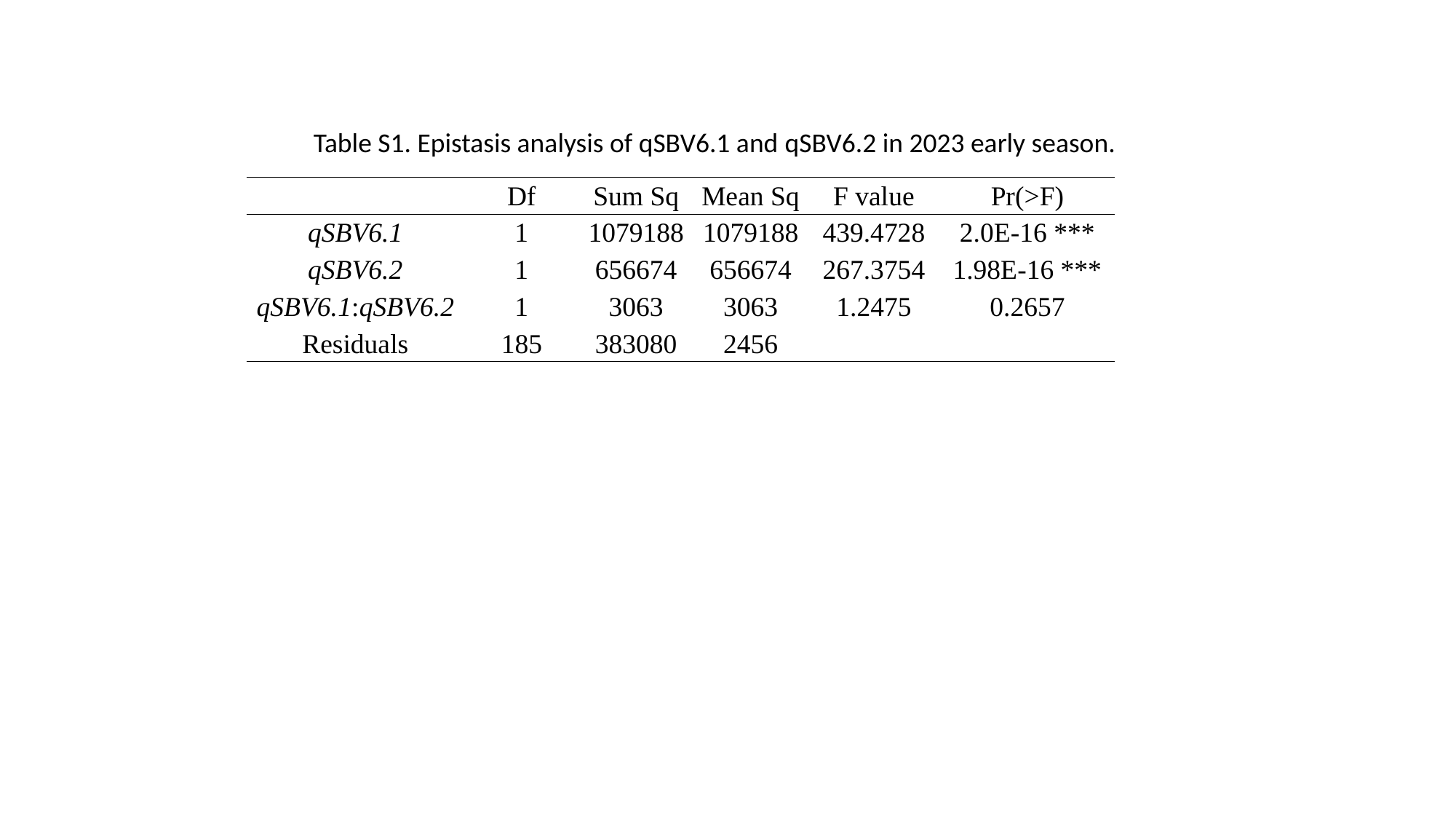

Table S1. Epistasis analysis of qSBV6.1 and qSBV6.2 in 2023 early season.
| | Df | Sum Sq | Mean Sq | F value | Pr(>F) |
| --- | --- | --- | --- | --- | --- |
| qSBV6.1 | 1 | 1079188 | 1079188 | 439.4728 | 2.0E-16 \*\*\* |
| qSBV6.2 | 1 | 656674 | 656674 | 267.3754 | 1.98E-16 \*\*\* |
| qSBV6.1:qSBV6.2 | 1 | 3063 | 3063 | 1.2475 | 0.2657 |
| Residuals | 185 | 383080 | 2456 | | |

## Slide 4
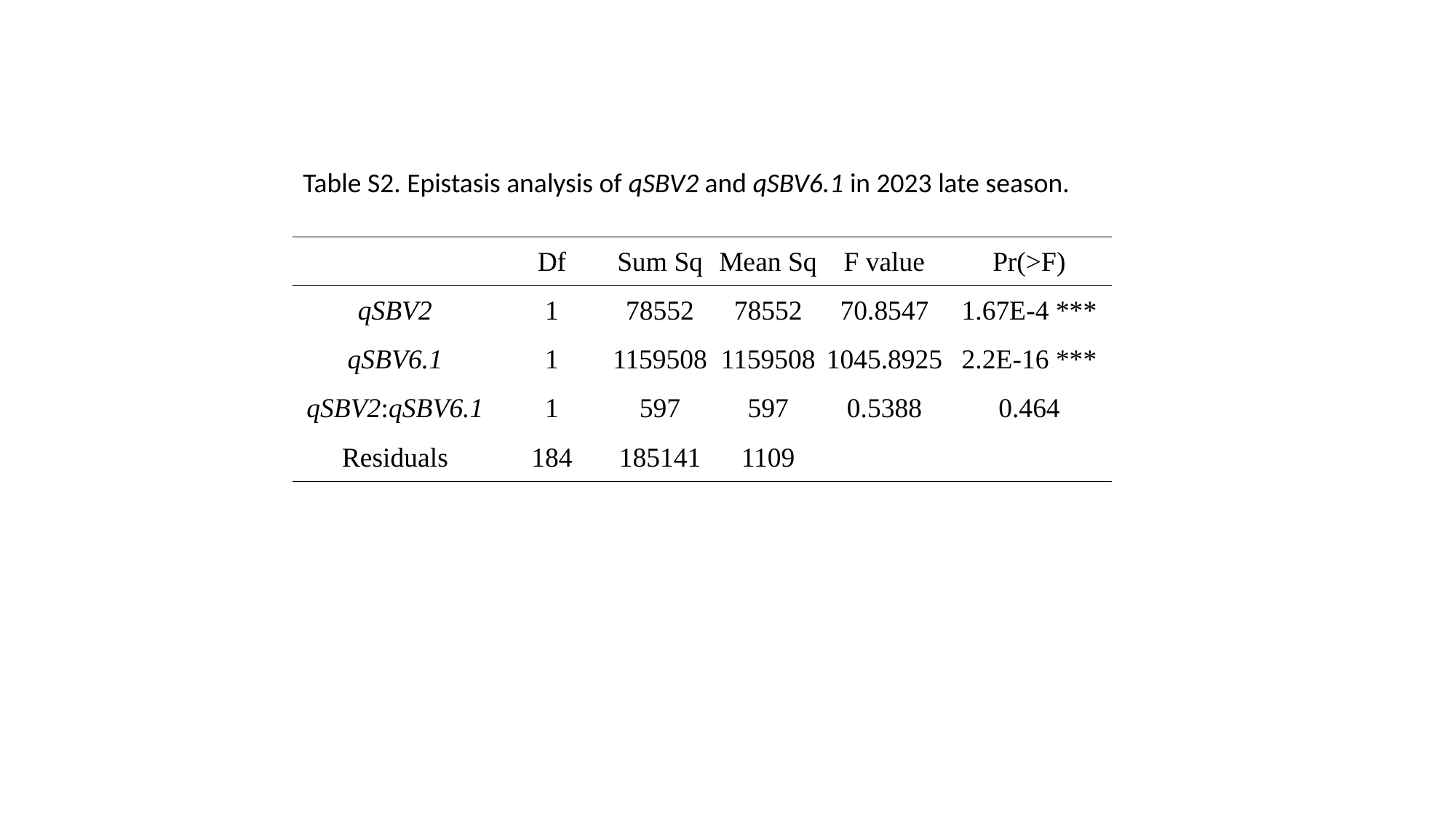

Table S2. Epistasis analysis of qSBV2 and qSBV6.1 in 2023 late season.
| | Df | Sum Sq | Mean Sq | F value | Pr(>F) |
| --- | --- | --- | --- | --- | --- |
| qSBV2 | 1 | 78552 | 78552 | 70.8547 | 1.67E-4 \*\*\* |
| qSBV6.1 | 1 | 1159508 | 1159508 | 1045.8925 | 2.2E-16 \*\*\* |
| qSBV2:qSBV6.1 | 1 | 597 | 597 | 0.5388 | 0.464 |
| Residuals | 184 | 185141 | 1109 | | |
